# Application of exposure bracketing to streamline the development of contraceptive products^☆^

**DOI:** 10.1016/j.conx.2022.100072

**Published:** 2022-01-30

**Authors:** Joshua Brown, Tamra Goodrow, Dan Hartman, Justin L. Hay, Kevin Hershberger, Susan Hershenson, Douglas McNair, Bethany Matthews, Mark A. Milad, Stephan Schmidt, Kirsten M Vogelsong, Ping Zhao

**Affiliations:** a College of Pharmacy, University of Florida, Gainsville, FL, United States; b Deer Run Regulatory Consulting, LLC, North Wales, PA, United States; c Bill & Melinda Gates Foundation, Seattle, WA, United States; d Medicines and Healthcare products Regulatory Agency, London, United Kingdom; e Certara, Princeton, NJ, United States; f Pharmica Consulting, Sparta, NJ, United States; g Milad Pharmaceutical Consulting LLC, Plymouth, MI, United States; h Department of Pharmaceutics, University of Florida, Orlando, FL, United States

**Keywords:** Contraceptive development, Exposure-bracketing, Levonorgestrel, Progestin, Pharmacokinetics, Real world data, BMGF, The Bill & Melinda Gates Foundation, FDA, US Food and Drug Administration, LNG, Levonorgestrel, MHRA, UK's Medical and Healthcare Products Regulatory Agency, PD, Pharmacodynamics, PK, Pharmacokinetics, RWDA, real world data analyses

## Abstract

Developing new long-acting products of well-characterized contraceptive drugs is one way to address some of the reasons for unmet need for modern methods of family planning among women in low- and middle-income countries. Development and approval of such products traditionally follow a conventional paradigm that includes large Phase 3 clinical trials to evaluate efficacy (pregnancy prevention) and safety of the investigational product. Exposure-bracketing is a concept that applies known pharmacokinetics and pharmacodynamics of a drug substance to inform its safe and efficacious use in humans. Several therapeutic areas have applied this concept by leveraging established drug concentration-response relationships for approved products to expedite development and shorten the timeline for the approval of an investigational product containing the same drug substance. Based on discussions at a workshop hosted by the Bill & Melinda Gates Foundation in December 2020, it appears feasible to apply exposure-bracketing to develop novel contraceptive products using well-characterized drugs.

## 1 Introduction

Millions of women in low- and middle-income settings want to avoid pregnancy but are not using a modern contraceptive method. A significant portion of women with this unmet need report method-related reasons for not using existing products [1]. The development of new contraceptive methods to address women's concerns and desires has been hampered by low levels of investment over the past several decades and can be considered a neglected global health issue [2,3]. Duration of action is an important consideration for progestin-based contraceptive users; some women seek long-acting products or products that would offer longer-term protection than current methods (e.g., a daily oral or 3-month injectable) but require less commitment than current three- of five-year implants.

Longer-acting reversible contraceptive products are preferred by many users for their ease of use, discreetness, and convenience. They also address adherence issues associated with daily oral use of contraceptive products that result in loss of effectiveness. Developing longer-acting products using a well-characterized and well tolerated drug substance can be expedient and efficient. There are decades of clinical efficacy and safety experiences and a wealth of information for contraceptive drug substances, including medroxyprogesterone acetate and levonorgestrel (LNG), that can be leveraged to inform the development of new products delivering these well-characterized compounds.

In December 2020, The Bill & Melinda Gates Foundation (BMGF) hosted representatives from multiple regulatory agencies and research organizations in a virtual workshop designed to investigate the robustness of the evidence base to support the applicability of an exposure-bracketing approach in the development of long-acting contraceptive products. This perspective summarizes major topics discussed at the workshop and is intended to inform broader contraceptive research communities on the utility and state-of-the-science of exposure-bracketing in contraceptive development.

## 2 Exposure-bracketing

### 2.1 Concept

Exposure-bracketing is a concept that applies known pharmacokinetics and pharmacodynamics of a drug substance to inform its safe and efficacious use in humans. The concept of exposure-bracketing is widely used throughout the continuum of drug discovery and development to support selection of dose and regimens. These applications include demonstration of therapeutic equivalence between a generic and a reference drug product, extrapolation of efficacy and dose modification in specific populations (e.g., pediatrics), management of clinical drug-drug interactions, and approval of alternative dosing regimens. When adequate information exists, exposure-bracketing may allow the use of pharmacokinetics as primary evidence for the approval of a newly formulated product of a well-characterized drug substance.

Pharmacokinetic and pharmacodynamic analyses are used to establish quantitative relationships between systemic exposure of a drug substance, i.e., drug concentrations in blood/plasma, and its efficacy and side effects in humans. These relationships are determined using data from previously conducted clinical trials to support product registration. Based on these relationships, one can derive a minimal effective concentration (i.e., an efficacy threshold) and a maximal safe concentration (i.e., a safety threshold), and bracket drug concentrations for an investigational product within these thresholds to inform the exposures that are associated with efficacious and safe use of the new product. The difference between the efficacy threshold and the safety threshold is also known as therapeutic window, safety margin, or therapeutic range.

**Fig. 1:**
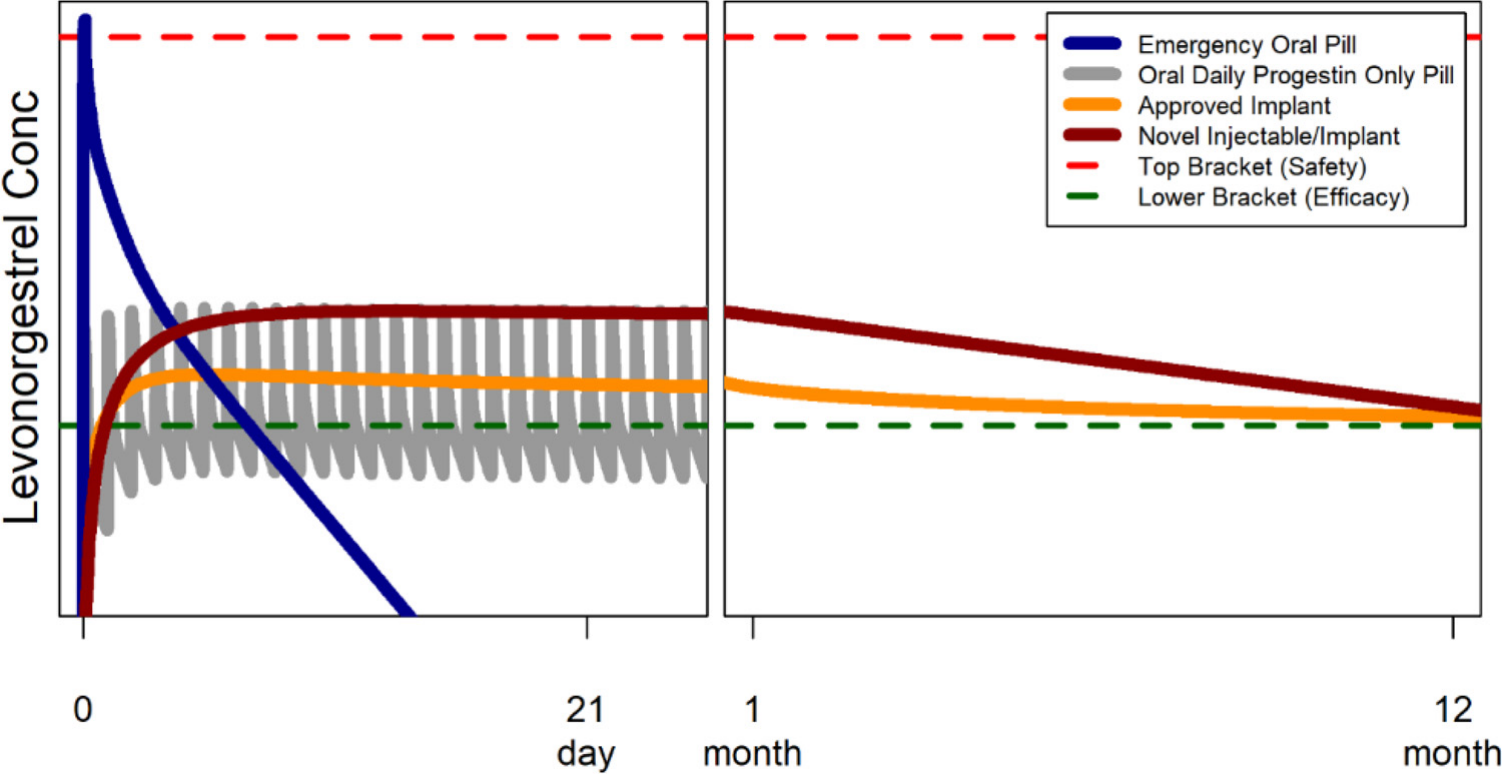
Application of exposure-bracketing to ensure safety and efficacy of a novel implant/injectable product of levonorgestrel, with levonorgestrel concentration presented in logarithmic scale (i.e., idealized data). Top and lower brackets are safety and efficacy thresholds that can be defined based on decades of clinical experience with various levonorgestrel products. For illustration purposes, if the levonorgestrel pharmacokinetic profile of the novel product is bracketed well within the thresholds (e.g., the fictional “Novel Injectable/Implant”), the product should be safe and efficacious for the intended duration (e.g., for 12 months). Oral daily only pill represented on this graph assumes the use of the lowest dose of levonorgestrel of 30 micrograms [4].

For example, Figure 1 shows an overlay of the varying pharmacokinetic profiles for several approved systemic LNG products. A new LNG product can be expected to be safe and effective if the systemic exposure of LNG is similar to or higher than the efficacy threshold observed following an approved, highly efficacious LNG-releasing subdermal implant, and substantially lower than the safety threshold established from the observed highest concentrations following various approved LNG oral products [4] with good safety profiles.

### 2.2 Vision to streamline the development of long-acting contraceptive products using exposure-bracketing

**Fig. 2:**
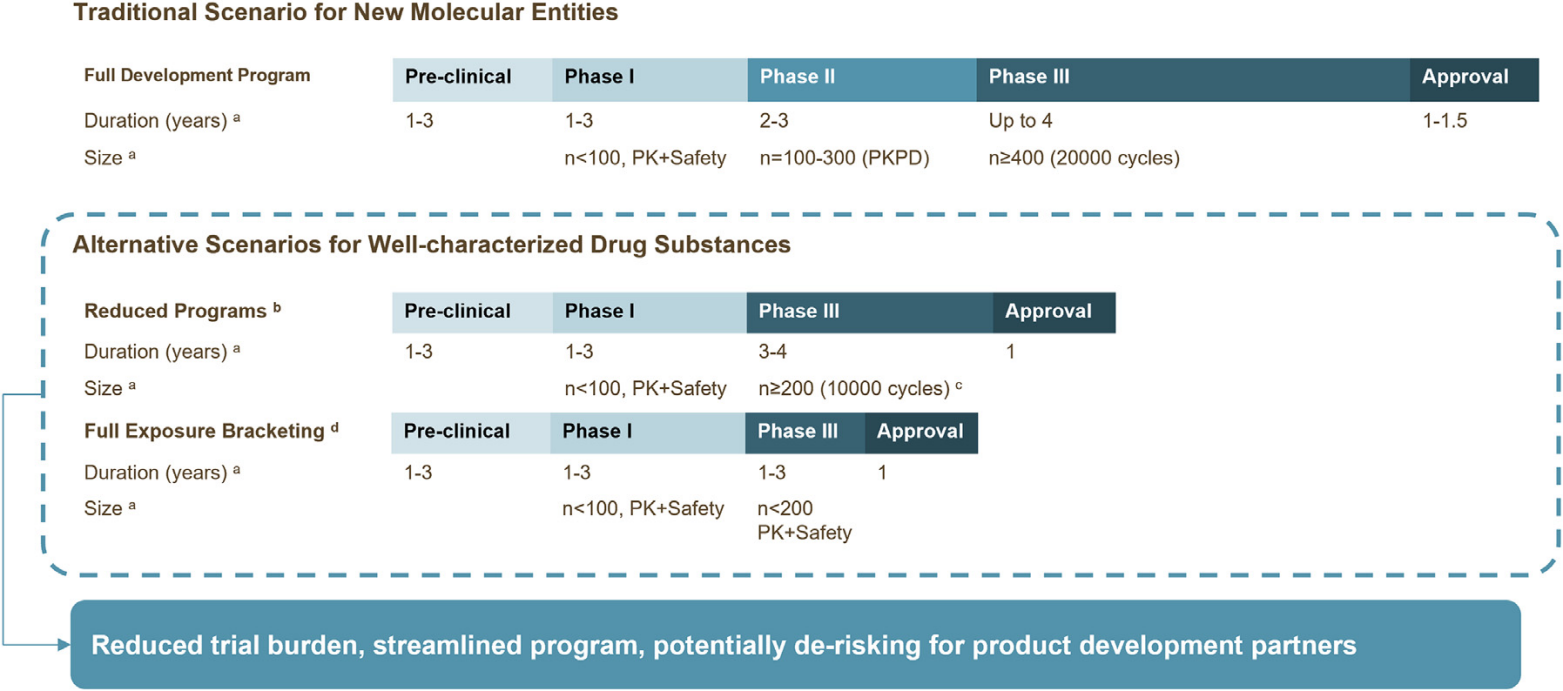
Alternative product development scenarios for products with well-characterized drug substances in contrast to the full development program traditionally designed for new molecular entities. ^a^Hypothetical estimates. A full development program to obtain approval by a stringent regulatory authority for a new molecular entity historically can take 10–15 years; ^b^ Reduced programs include an abridged pathway in the European Union and a 505b(2) pathway in the US; ^c^ Captured in recent US FDA draft guidance^[^^12^^] d^ Full exposure bracketing approach maximally leverages existing knowledge of a drug substance to further shorten the clinical program by reducing the size and focusing on evaluating safety specific to the new product (e.g., the performance of a novel formulation in a phase III trial). Overall duration of follow-up in a phase III trial will depend on the targeted duration of the new product. Abbreviations: PK = pharmacokinetics; PD: pharmacodynamics.

If prior knowledge of a well-characterized contraceptive drug substance from approved products is not leveraged, the development of a new long-acting formulation of the same contraceptive drug substance typically would follow the conventional drug development pathways that are required for new molecular entities: conducting a full clinical program, with multiple large trials, to evaluate efficacy and safety of a new formulation (Traditional scenario, Fig. 2) [5,6]. Figure 2 shows alternative development pathways applying the exposure-bracketing concept for novel contraceptive products with well-characterized progestins. The first alternative scenario (Reduced Program) follows the currently accepted abridged program [7] in Europe and the 505(b)(2) pathway [8] in the US. The reduced program by-passes phase II dose-range finding studies intended to demonstrate efficacy, and directly progresses into confirmative phase III trials. The second alternative (Full Exposure-Bracketing) leverages existing knowledge from approved products to further streamline the pivotal program of an investigational product. This approach primarily uses the pharmacokinetics of approved products to extrapolate efficacy and safety; consequently, the clinical program focuses on evaluating safety specific to the newly formulated product. For example, if the new product is a subcutaneous injectable, the pivotal trial would be designed to evaluate safety at the site of the injection, systemic safety of the drug substance would be extrapolated from the approved products if the overall systemic exposures are similar between the approved products and the investigational product. The size and duration of the pivotal trial then depends on the confidence in the bracket. Prior applications of exposure-bracketing have expedited the development of several long-acting products [9]. For example, in psychiatry, paliperidone 3-month injectable received approval partially based on exposure-response relationships established for approved 1-month injectable products, with a much shorter development timeline and a relatively small clinical development program [10].

For contraceptive products, key considerations to develop criteria for using an exposure-bracketing approach include pharmacokinetic characteristics for safety and efficacy of a drug substance, tools that have been used to capture efficacy (e.g. suppression of ovulation, Pearl Index [11,12]), impact of variability and quality of data generated for approved products, selection of one or more approved products as active comparators, and mathematical models that can comprehensively describe pharmacokinetics and pharmacodynamics of drug substance of approved products some potentially with differing routes of administrations. If a pharmacokinetic study becomes the pivotal study, including an active comparator may be useful when comparing results across different studies (historical controls).

Effective application of exposure-bracketing benefits from an integrated modeling framework connecting various methodologies [13]. A rigorous framework includes:

1 Model based meta-analysis: quantification of dose-response relationships by pooling data from different studies;
2 Physiologically based pharmacokinetic models: mathematical description of human physiology and its interactions with drug molecules to translate dose-response relationships into respective exposure-response relationships in a specific population of interest; and
3 Real world data analysis: analysis of data “relating to patient health status and/or the delivery of health care routinely collected from a variety of sources” [14], using regression modeling and/or machine-learning methods to detect the absolute or relative clinical signal and a propensity score method to reduce measured confounding, bias, and heterogeneity [15,16]

## 3 Levonorgestrel as a model contraceptive agent for exposure-bracketing

Application of the integrated modeling framework described above helps to address challenges and caveats related to leveraging data of approved LNG products to inform the development of new LNG products. These caveats include variability in LNG's pharmacokinetics and pharmacodynamics; a relatively high fluctuation in LNG concentrations after oral administration; the effect of estrogen in combination products on the pharmacokinetics and efficacy of a given dose of LNG; the relatively fewer doses studied for LNG implants; the value of historical pharmacokinetic data measured using older methodologies (i.e., radioimmunoassay); and the effect of specific user factors (e.g., body mass index, adherence, concomitant medications).

Cicali et al. recently used physiologically based pharmacokinetic (and pharmacodynamic) models to delineate a wide range of LNG concentrations in the presence of complex dynamic interplay with ethinyl estradiol, sex hormone binding globulin, albumin, and concomitant medications in both non-obese and obese women [17]. Coupled with model-based meta-analysis that describes the quantitative relationships between trough LNG concentrations at steady state and Pearl Index in women taking oral pills, the model enables prediction of the effect of concomitant medications on LNG efficacy [18]. Analyzing real-world outcomes data from FDA's Adverse Event Reporting System, Sunaga et al. showed that varying routes of administration can influence the magnitude of drug-drug interactions for the same progestin when women concomitantly take a cytochrome P450 3A inducer [19]. An administrative claims database analysis by Sarayani et al. found differences in pregnancy rates between women using combined oral contraceptives concomitantly with anti-epileptic drugs with and without potential to induce cytochrome P450 3A [20].

### 3.1 Defining the bracket for efficacy

An efficacy threshold for contraception can be derived from extensive systemic pharmacokinetic data of approved LNG products and supportive exposure-response analyzes. Circulating total systemic LNG concentrations of >200 picogram per milliliter (pg/ml), in an individual would likely ensure an acceptable contraceptive protection [6]. This efficacy threshold was confirmed by the exposure-efficacy relationship of Jadelle and Sino-implant (now Levoplant), two versions of LNG-releasing contraceptive impants [21]. Of the 1393 women who received Jadelle in the product's registration trial, 3 became pregnant by the end of 48 months [6]; of the 136 women receiving Jadelle in a more recent clinical trial of Sino-implant, no pregnancy was observed by the end of 48 months [21]. In this newer trial, the authors reported significantly higher pregnancy rate in the fourth year (48 months) compared to the first 3 years combined for Sino-implant (3.54 pregnancy per 100 women-year versus 0.18 per 100 women-year, respectively). At the end of 48 months, The total mean plasma concentrations of LNG in Sino-implant users appear to be lower than that in Jadelle users (205 pg/mL vs 299 pg/mL) [21].

Although one must acknowledge the differences in assay methodologies and specimens in which LNG was analyzed when evaluating pharmacokinetic studies conducted over decades of research [22], LNG concentrations reported from the aforementioned trials in women using Jadelle were generally similar. The mean *serum* levels of LNG measured using *radioimmunoassay* at the end of months 1, 12, 24, 36, and 48, as reported in Jadelle's package insert, were 435, 340, 312, 280, and 271 pg/ml, respectively [6]; the corresponding mean *plasma* drug levels measured using *liquid chromatography-tandem mass spectrometry assay* in the newer trial were 453, 314, 310, 276, and 299 pg/ml, respectively [21]. In comparison, the mean *plasma* levels reported for Sino-implant were 428, 310, 252, 220 and 205 pg/ml, respectively.

As such, if an individual maintains a total plasma concentration of LNG greater than 200 pg/mL over the intended use period of an investigational injectable or implant, an acceptable contraceptive protection should be expected. This individual efficacy threshold of 200 pg/mL is further supported by model-based meta-analysis of clinical trials for orally administered LNG pills [18]. The analyses derived a model that describes the relationship between trough LNG concentration at steady state and pregnancy prevention measured by Pearl Index. For example, the mean trough concentrations of 410, 248, and 136.5 pg/mL are related to Pearl Indices of 2, 3, and 5, respectively.

Due to foreseeable interindividual variability in pharmacokinetics of LNG for any given product, the developer of an investigational LNG product can set a mean efficacy threshold greater than 200 pg/mL, so that the vast majority of individual subjects maintain a concentration of 200 pg/mL or greater. For example, if pharmacokinetic variability unique to this investigational product is estimated in Phase 1 safety trial to be similar to that observed for Jadelle, one can target a more conservative mean value of 279 pg/mL reported for Jadelle users at the end of 60 months as a practical efficacy threshold [6]. If the variability is greater than Jadelle then the corresponding target mean concentration would also need to be greater.

### 3.2 Defining the bracket for side effects

The determination of the safety exposure threshold of a drug is more complex because the threshold varies depending on the side effect of concern. Compared to efficacy-related endpoints, exposure-response relationship analyses for safety of progetin-based contraceptives are more limited due to their excellent safety profiles over a wide exposure range and the rare occurrence of safety events. For well-characterized progestins such as LNG, real-world data analyses may play a critical role in defining the safety thresholds by complementing and evaluating the dose/exposure-response analyses in more generalizable patient populations receiving routine clinical care.

The determination of safety thresholds should consider the following:

a Good safety profiles of progestins. Progestin-only contraception is considered safe as most serious adverse reactions associated with contraceptive steroids are related to estrogen, for example in the combined hormonal contraceptives. Bone density loss and fracture potential have been concerns primarily with medroxyprogesterone acetate injections, but not with LNG and etonogestrel subdermal implants. Over the decades of use, no serious safety signals have emerged for LNG implants. Irregular bleeding is an important factor in user satisfaction and can affect continuation and potential uptake of a contraceptive drug product. Understanding the quantitative relationships of progestin exposure and bleeding profiles would inform one aspect of the side effect threshold for a new long-acting LNG-only drug product.
b Validity and relevance of real-world data generated in high income setting. Most real-world data analyses are derived from databases in high income countries, with varying populations represented based on health insurance coverage. Generalizability of these results to women from other regions should be confirmed. In a real-world setting, poorer adherence (i.e., typical use) makes it difficult to establish exposure-response relationships for side effects by diluting the signals but provides longitudinal follow-up of large populations to identify rare outcomes and subpopulations that may be at increased risk of side effects due to demographic, health or other factors.
c Difference in pharmacokinetic profiles from products with different routes of administration (e.g., daily oral versus long-acting implants). The analyses to translate the exposure-response relationship for safety from one product to another is complicated by the different LNG pharmacokinetic profiles. This requires the use of integrated quantitative modeling methods mentioned above to help bridge the gap [13,18].

## 4 Regulatory and development considerations with respect to using active comparator(s) in clinical trials

Besides leveraging historical knowledge and experience of approved products, development of a novel contraceptive product of a well-characterized drug substance may benefit from including an approved product as an active comparator to support the use of exposure-bracketing approach. The developer should address the following questions when deciding whether a comparator should be used:

a What value will additional data generated from comparator arm provide to the current understanding of clinical performance of a contraceptive drug substance?
b If a bracket is acceptable for efficacy and safety based on robust historical data for a given drug substance, is a comparator(s) still needed?

Whether or not to include a comparator depends on (i) the similarity between the comparator and the investigational product with respect to formulation, route and frequency of administration, (ii) the availability of a relevant formulation of the comparator, (iii) the regulatory pathway selected, and (iv) the robustness of available or historical pharmacokinetic data to support bracketing for safety and efficacy.

An investigational long-acting product may differ from the approved products in terms of intended duration of use. Developers may consider using more than one approved product as comparators or truncating the duration of an approved product with longer duration to match that of the investigational product with a shorter duration. From an efficacy standpoint, including an active comparator will be more critical if the pharmacokinetics of progestin of an investigational product approach the established thresholds. If historical data were generated using different analytical methods or are otherwise not considered to be sufficient to define bracketing, a comparator might then be needed in the pharmacokinetic study of the investigational product to support a more expedient pathway to regulatory approval.

Agreeing on an exposure bracket for a given progestin is complex and can be compared to the qualification of a biomarker for drug development purposes. If qualified and broadly agreed by the community, including regulators, the proposed exposure bracket of a specific progestin (e.g., the mean efficacy threshold of either 200 or 280 pg/mL for LNG) can be applied by the developer without re-justifying it every time. It is recommended for developers to have early dialog with regulators to make the scientific case on why the use of historical data may be adequate and the inclusion of a comparator may or may not be warranted in a development program.

## 5 Conclusion and perspectives

The majority of workshop attendees agreed that establishing a bracket for well-characterized contraceptive progestins such as LNG is achievable for efficacy. Establishing a bracket for safety was also generally agreed to be achievable. However, the safety assessment is more complex given the range of side effects associated with the use of contraceptive drug products and potential variability in exposure profiles with different formulations and delivery methods. To this end, consensus is needed on how to associate safety and side effects with exposure data, including the analyses of real-world data in addition to clinical trial data. There was a general agreement that a bracketing approach could streamline the development program of an investigational product. The use of a comparator is product- and regulatory pathway-specific and should be discussed with regulatory agencies during development for alignment. The science in understanding the exposure-response relationship of progestins has advanced to allow the use of exposure-bracketing to streamline product development for well-characterized progestin contraceptives, an area that has been relatively neglected in the past.

## Disclaimer

The content of this report does not reflect the views or policies of the US Food and Drug Administration (FDA), UK Medicines & Healthcare products Regulatory Agency (MHRA), Bill & Melinda Gates Foundation, or their staff. No official support or endorsement by the FDA or Bill & Melinda Gates Foundation is intended or should be inferred.

## Declaration of Competing Interest

The authors declare no conflict of interest.

## Figures and Tables

**Table:** 

Name	Affiliation	Role
Nicole Assmann	U.K. Medicines & Healthcare products Regulatory Agency	Panelist
Eva Gil Berglund	Certara	Moderator
Carin Bergquist	Swedish Medical Products Agency	Panelist
Swati Bhat	U.K. Medicines & Healthcare products Regulatory Agency	Panelist
Joshua Brown	University of Florida	Presenter
Julie Bullock	Certara	Panelist
Susan Cole	U.K. Medicines & Healthcare products Regulatory Agency	Panelist
Tamra Goodrow	Deer Run Regulatory Consulting	Panelist
Dan Hartman	Bill & Melinda Gates Foundation	Presenter
Justin Hay	U.K. Medicines & Healthcare products Regulatory Agency	Presenter
Kevin Hershberger	Certara	Moderator
Susan Hershenson	Bill & Melinda Gates Foundation	Presenter
Joachim Hochel	Bayer AG	Panelist
Ian Hudson	Bill & Melinda Gates Foundation	Moderator
Steven Kern	Bill & Melinda Gates Foundation	Panelist
Dennis Lee	Bill & Melinda Gates Foundation	Moderator
Hanbin Li	Certara	Panelist
Murray Lumpkin	Bill & Melinda Gates Foundation	Moderator
Douglas McNair	Bill & Melinda Gates Foundation	Presenter
Mark Milad	Milad Pharmaceutical Consulting	Presenter and Panelist
Raajan Naik	U.S. Food and Drug Administration	Presenter
Stephan Schmidt	University of Florida	Presenter
Shirley Seo	U.S. Food and Drug Administration	Presenter and Panelist
Elizabeth Smith	Bill & Melinda Gates Foundation	Moderator
Doanh Tran	U.S. Food and Drug Administration	Panelist
Kirsten Vogelsong	Bill & Melinda Gates Foundation	Moderator
Carolyn Westhoff	Columbia University	Panelist
Gerald Willett	U.S. Food and Drug Administration	Panelist
Ping Zhao	Bill & Melinda Gates Foundation	Moderator
